# Human factor associations with safety events in radiation therapy

**DOI:** 10.1002/acm2.13420

**Published:** 2021-09-10

**Authors:** Sheri M. Weintraub, Bill J. Salter, C. Lynn Chevalier, Sarah Ransdell

**Affiliations:** ^1^ Department of Radiation Oncology Southcoast Centers for Cancer Care, Southcoast Health Fairhaven Massachusetts USA; ^2^ Department of Radiation Oncology Huntsman Cancer Institute University of Utah Salt Lake City Utah USA; ^3^ Dr. Pallavi Patel College of Health Care Sciences Nova Southeastern University Fort Lauderdale Florida USA

**Keywords:** human factors, incident learning, patient safety, radiation oncology, radiation therapy

## Abstract

**Background and purpose:**

Incident learning can reveal important opportunities for safety improvement, yet learning from error is challenged by a number of human factors. In this study, incident learning reports have been analyzed with the human factors analysis classification system (HFACS) to uncover predictive patterns of human contributing factors.

**Materials and methods:**

Sixteen hundred reports from the Safety in Radiation Oncology incident learning system were filtered for inclusion ultimately yielding 141 reports. A radiotherapy‐specific error type was assigned to each event as were all reported human contributing factors. An analysis of associations between human contributing factors and error types was performed.

**Results:**

Multiple associations between human factors were found. Relationships between leadership and risk were demonstrated with supervision failures. Skill‐based errors (those done without much thought while performing familiar tasks) were found to pose a significant safety risk to the treatment planning process. Errors made during quality assurance (QA) activities were associated with decision‐based errors which indicate lacking knowledge or skills.

**Conclusion:**

An application of the HFACS to incident learning reports revealed relationships between human contributing factors and radiotherapy errors. Safety improvement efforts should include supervisory influences as they affect risk and error. An association between skill‐based and treatment planning errors showed a need for more mindfulness in this increasingly automated process. An association between decision and QA errors revealed a need for improved education in this area. These and other findings can be used to strategically advance safety.

## 1 INTRODUCTION

Preventable errors brought about by human contributing factors are on par with some of the most notable diseases in terms of their negative impact on human health.^1^ In the field of radiation oncology (RO), incident learning provides an opportunity to prevent the recurrence of some of those errors. Incident learning systems (ILSs) are communication forums that allow errors to be used as opportunities for future risk reduction. The RO‐ILS is available for this purpose in the United States and the International Atomic Energy Agency's Safety in Radiation Oncology (SAFRON) system is available to the international radiotherapy community.^2^, ^3^

Incident learning has its roots in the aviation industry. Following a 57% decrease in the US accident rate, aviation was recognized as a high‐reliability organization, one that is complex and hazardous yet has achieved long‐standing records of safety.^4^, ^5^ Healthcare is also complex and hazardous yet has not yet achieved that status of high reliability.

Studies of aviation accidents revealed that human contributing factors to error were partially responsible in over 70% of cases.^6^ The human factors analysis classification system (HFACS) was developed to analyze and classify these factors.^7^ The goal of this study was to use the HFACS to find predictive patterns of human factors in SAFRON incident learning reports. Several important associations were uncovered as part of this work.

## 2 METHODS

### 2.1 Data selection

Incident learning reports from the SAFRON system were obtained with permission from the International Atomic Energy Agency.^3^ Over 1600 reports in this database were filtered for information inclusion. Included reports contained contributing factors and a descriptive free text narrative. Reports were excluded if a) there was no response to the prompt “describe the discrete causes of the incident” or if b) they were of lower or unspecified severity. Event severity was chosen by the event submitter from a menu of SAFRON options which included a “minor incident” category containing minor actual and near‐miss events. These were all excluded. Included options had more severe descriptors and also included near‐miss versions of those events. The final data set contained 141 reports.

All reports were rated as having either lower, medium, or high‐quality information. While all reports met the minimum standard for data inclusion, some allowed for a clear and detailed understanding of the safety event and some did not. For example, a lower quality report provided minimal qualitative information beyond the required discrete data and a one or two‐sentence narrative. A high‐quality report offered lengthy detail regarding what happened, who was involved, and perceived root causes. A rubric defining these three levels of information value supported a consistent quality assessment. This, in turn, was used to ensure that report quality did not influence study findings.

### 2.2 Error type and human contributing factor assignments to reports

**FIGURE 1:**
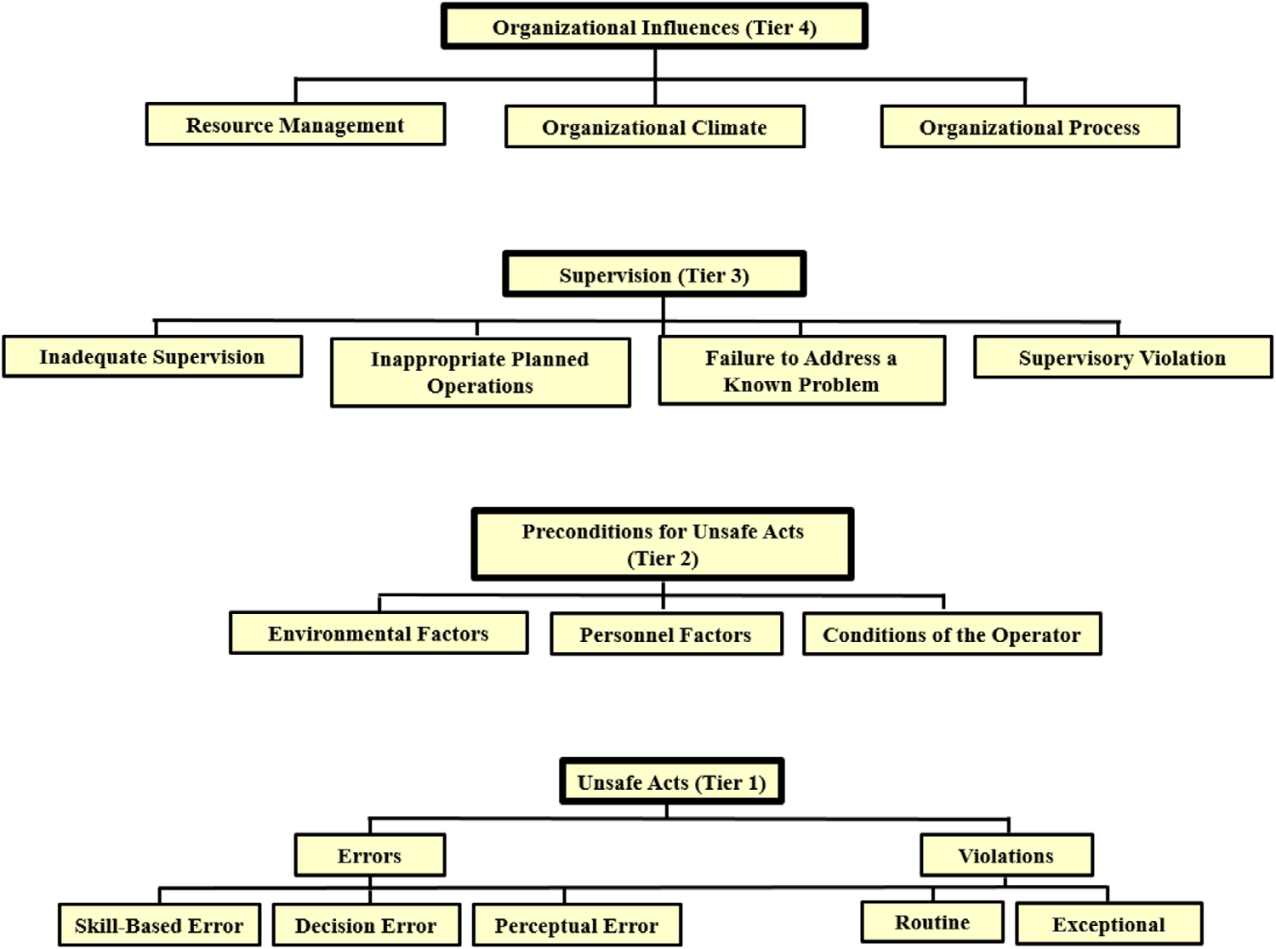
The human factors analysis classification system. Note: Four tiers of human factors represent distinct categories of contributing factors to error as adapted from Diller et al.^9^

Each incident report was assigned a single error type that best described what happened (Table 1).^8^ Each incident report was also assigned human contributing factors from the HFACS tool. The HFACS contains four tiered categories of human contributing factors as well as a number of more specific subcategories (Figure 1).^9^ The first tier, unsafe acts, contains five subcategories of errors or violations that resulted in an undesirable outcome. The second tier, preconditions for unsafe acts, contains three environmental conditions that contributed to increased risk. The third tier, supervision, contains four distinct kinds of supervisory failures that relate to training, guidance, and general oversight. The fourth tier, organizational influences, contains three kinds of upper‐level management failures that affected supervisors and staff. All HFACS subcategories mentioned in a report were assigned to that report. As incident reports did not necessarily mention a subcategory from the HFACS verbatim, each subcategory was clearly defined (Table 2) so that it could be consistently mapped. Human contributing factor terms from the incident learning reports were consistently mapped to human contributing factor terms from the HFACS.

**TABLE 1:** Radiation therapy error types

**Error type**	**Description**
Approval	Appropriate approval was missing or something was inappropriately approved
Documentation incomplete	Documentation was incomplete or missing
Documentation erroneous	Documentation contained erroneous or inaccurate information
Equipment	Equipment malfunction included software, hardware, connectivity, or networking
Image guidance	Images were not interpreted correctly, shifts were made in the wrong direction or by an incorrect amount based on a misreading of images, images were accidentally omitted or too many images were taken, image guidance instruction was not followed, there was a failure to assess images based on priority instruction, or there was a lacking skillset in analyzing images appropriately
Treatment planning	The planner used the wrong dose, wrong technique, planner did not account for certain anatomy or implants, prior RT was disregarded, an incorrect target or contours were used, the wrong CT data set was used, etc.
Collision	Either a collision or a potential collision with equipment and/or a patient
Quality assurance	Quality assurance of a treatment plan, ongoing treatment, or equipment was performed erroneously, was missed, missed a standard component, or was suboptimal with respect to applicable policy or guidelines
Scheduling	Any error around patient scheduling
Simulation	Wrong body part or location simulated, substandard scan protocol, insufficient scan length, incorrect isocenter marked or tattooed, failure to complete the standard simulation process
Treatment setup	These errors include setup and positioning errors, alignment to incorrect skin markings, forgotten or misplaced bolus, incorrect shifts in setting up the patient for treatment, etc.
Treatment delivery	These errors include the wrong field treated, a missing treatment accessory, or any other issue that involves an incorrect component of radiation delivery (not setup)
Wrong patient	Patient ID issue
Patient	A patient‐related problem occurred such as a significant delay, miscommunication, health problem unrelated to treatment, inadequate coordination with their other care providers, or failure to comply with the information in their medical record outside radiation therapy such as allergies or other special care needs

**TABLE 2:** Human factor descriptions from the human factors analysis classification system

**Human factors (HFACS tier and subcategory)**	**Description**
**Tier 1 Unsafe Acts**	**These are the actual actions that take place resulting in an undesirable outcome**
Skill‐based errors	Errors made when performing familiar tasks that are routinely done without a great deal of thought
Decision errors	Information, knowledge, or experience is lacking
Perceptual errors	Input to any of the five senses is compromised and someone subconsciously fills in missing information
Routine violations	Disregard for the rules but habitual in nature as "bending the rules" is typically tolerated
Exceptional violations	Disregard for the rules in a way that is atypical, not done by others, and not condoned by leaders
**Tier 2 Preconditions for unsafe acts**	**This describes the environment and conditions contributing to increased risk**
Environmental factors	Physical Environment: Issues with the physical environment such as poor ergonomic layout, lighting, or clutter OR Technical Environment: Issues with equipment, networking, the human‐computer interface, or automation
Personnel factors	Communication: miscommunication between individuals ‐ information either unavailable or incomplete OR Coordination: healthcare providers work independently and do not manage care well between them OR Planning: patients’ needs are not correctly anticipated or for any reason, appropriate care plans are not made OR Fitness for Duty: Care providers have a substandard physiologic state due to issues such as being tired or being on medication
Operator conditions	Adverse Mental State: Examples include fatigue, stress, or distraction OR Adverse Physiological State: Examples include illness, injury, or other temporary incapacitation OR Chronic Performance Limitation: These limitations are either chronic or long term
**Tier 3 Supervision**	**These are issues with front line management who are directly responsible for training, guidance, and oversight**
Inadequate supervision	Leadership failure with respect to training, guidance, and keeping up with best‐practice standards
Inappropriate planned operations	Includes schedule, assigning duties, and keeping staff aware of the plan so that they are able to execute
Failure to address a known problem	Applies when deficiencies, equipment failures, a lack of training, or other issues are known and ignored
Supervisory ethics violation	Applies when supervisors allow things to occur that are known to be against policy or regulations
**Tier 4 Organizational Influences**	**These involve upper‐level management decisions that affect supervisors and staff**
Organizational resource management	Resource allocation and maintenance including budgets, equipment, and staffing allowances
Organizational climate	Describes the safety culture, working atmosphere, a chain of commands and values
Organizational process	Includes failures of corporate rules that govern everyday activities such as scheduling and communication

### 2.3 Associations between human contributing factors

As defined within the HFACS framework, unsafe acts are the most directly linked to a safety event. Human factor classifications become progressively more indirect with preconditions for unsafe acts and supervisions, and organizational influences have the most indirect association. After assigning all reported human contributing factors (HFACS subcategories) to the incident reports, associations between less and more directly related human factors were sought. Testing was done for associations between higher and lower‐tiered factors from the HFACS. The goal was to determine how human contributing factors relate to one another, specifically how factors more remote to unsafe acts may predict the likelihood of more directly involved factors. With this, we can improve organizational, supervisory, and environmental safety and reduce the risk of unsafe acts.

Associations between human contributing factors were calculated with Goodman and Kruskal's lambda test (SPSS v22) to determine the proportional reduction in error (PRE) between higher and lower tier HFACS subcategories. This test had been utilized similarly for aviation safety event studies.^10^, ^11^ A statistically significant association indicates that a human contributing factor more directly related to the safety event could be more reliably predicted if the more remote human contributing factor was known. Lambda (PRE) values below 0.1, between 0.11 and 0.30, and above 0.31 indicate weak, moderate, and strong associations respectively. Odds ratios were calculated for each significant finding.

### 2.4 Associations between human contributing factors and error types

Testing was also done to determine whether there were significant relationships between human contributing factors and radiation therapy error types. Chi‐square tests for independence were run with each of the human contributing factors (HFACS subcategories) and all 15 error types. Adjusted residuals were used to determine which error type was related to the human factor and odds ratios were again calculated for significant findings.

## 3 RESULTS

Forty‐nine percent of the SAFRON reports were classified as high quality with strong descriptive detail. Thirty‐three percent were of medium quality, and 18% were of lower quality. Descriptive statistics were analyzed for incident learning reports both with and without the inclusion of the lower quality reports. There were no significant differences found and all 141 reports were included in the study.

**FIGURE 2:**
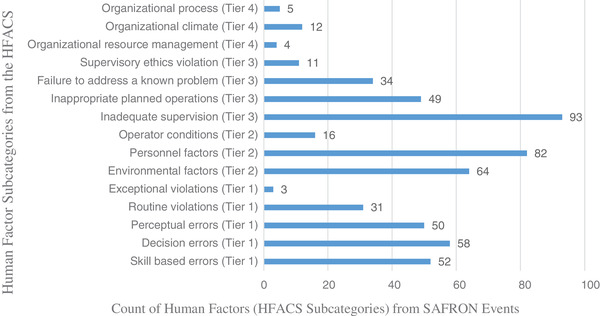
Prevalence distribution of human contributing factors. Note: This graph shows the distribution of 564 human contributing factors from all four tiers of the human factors analysis classification system (HFACS). The assignments were distributed among 141 individual Safety in Radiation Oncology (SAFRON) event reports

A total of 564 human contributing factors (subcategories from the HFACS) were mentioned in the 141 incident reports. There was at least one human contributing factor mentioned in each report and as many as eleven. There were likely additional contributing factors that were not mentioned by SAFRON event submitters but study data was comprised only of what the submitters had included. Among the four tiers of the HFACS, 34% of reported human contributing factors fell under unsafe acts, 29% fell under preconditions for unsafe acts, 33% fell under supervision, and 4% fell under organizational influences (Figure 2).

PRE was calculated to find associations between human contributing factors to safety events. PRE calculations revealed relationships between unsafe acts and less directly involved factors: preconditions for unsafe acts, supervisory influences, and organizational influences. Four significant relationships were found (Table 3). An association was found between inadequate supervision and decision errors (lambda value of 0.25), suggesting that when there is inadequate supervision there is a 25% increased likelihood of also having decision errors. Decision errors were over six times as likely to be reported when inadequate supervision had also been reported (odds ratio = 6.26). Predictive associations between radiotherapy error types and human contributing factors were evaluated with the chi‐square test for independence and several significant relationships were found (Table 4). An association between treatment planning and skill‐based errors suggests that treatment planning safety events are influenced by slips made without a great deal of thought while routine tasks are being performed. An association between decision and quality assurance (QA) errors suggests that safety events involving QA work are influenced by lacking knowledge or skill.

**TABLE 3:** Significant associations between human contributing factors to error

**Human contributing factor subcategories from the HFACS**				
**Higher‐tier factor**	**Lower‐tier factor**	**Lambda (PRE)**	***p*‐value**	**Odds ratio**
Tier 3 SIS	Tier 1 ED	.25	.08	6.26
Tier 3 SFP	Tier 1 ED	.14	.16	3.95
Tier 3 SFP	Tier 2 PE	.12	.16	2.80
Tier 3 SFP	Tier 2 PP	.13	.07	0.37

*Note*. This table displays higher‐tier subcategories from the human factors analysis classification system (HFACS) that are associated with lower‐tier subcategories. Goodman and Kruskal's lambda values less than 0.10 are considered weak associations, values 0.11 to 0.30 are considered moderate, and values greater than 0.31 are considered strong. Tabled associations have a minimum of five SAFRON events in each cross‐tabulated category and a *p*‐value of up to .20. Abbreviations for subcategories from the HFACS are listed in Table 4.

**TABLE 4:** Significant associations between error type and human contributing factors to error

**HFACS tier and subfactor code**	***Χ* ^2^**	***p*‐value**	**Associated error 1**	**Adjusted residual 1**	**OR 1**	**Associated error 2**	**Adjusted residual 2**	**OR 2**
Tier 1 unsafe acts, ES	26.88	.01	Treatment planning	2.1	2.33			
Tier 1 unsafe acts, ED	52.86	<.01	QA	5.5	25.0			
Tier 1 unsafe acts, EP	24.67	.03	Image guidance	2.0	3.67			
Tier 2 preconditions for unsafe acts, PE	24.84	.02	Equipment	2.4	5.81	Image guidance	2.4	5.81
Tier 3 supervision, SFP	26.67	.01	Image guidance	2.8	5.56	QA	2.6	3.74

Abbreviations: HFACS, human factors analysis classification system; OR, odds ratio; QA, quality assurance.

*Note*. Significant relationships between error types and human contributing factors are listed if their chi‐square value is significant with a *p‐*value less than .05, with an *n* greater than 5, and with an adjusted residual greater than 1.96.

## 4 DISCUSSION

Human factors contribute to errors in radiation therapy and have multiple predictive associations. Supervision failures significantly impact the likelihood of unsafe acts. Leadership includes oversight of training, education, and best practice standards that are directly tied to safety. One specific kind of human contributing factor, skill‐based error, leads to treatment planning errors. Skill‐based errors are those made while performing familiar tasks without a great deal of thought. We also found that decision errors, those made purposefully without proper knowledge, are associated with QA errors. Safety improvements with QA work should therefore be addressed through improved training and education.

The HFACS was used successfully in this study and in previously published studies to analyze human factor contributions to safety events.^12^ The tool helps organize a focus on why those events took place as opposed to what happened and who was involved.^13^ Through this human factors‐based approach, we found specific associations within the SAFRON safety event database. These associations can be used to inform targeted safety improvement efforts. Our finding is in contrast to other investigators who did not use the HFACS and concluded that incident learning databases lack the detail needed to support that kind of effort.^14^, ^15^

The association between latent supervisory errors and other safety events has not been corroborated in radiotherapy‐specific publications, but similar associations have been found elsewhere. Organizational function, with an indirect influence on safety much like that of supervision, has been shown to impact errors and mishaps.^16^ Supervision was specifically shown to impact human performance and accidents in the field of aviation.^17^

The association found between treatment planning and skill‐based errors (or mindless slips) is also uniquely specific to radiation therapy. Other studies demonstrate improved safety through automation but those improvements do not include contributing factors related to human influence.^18^, ^19^ The association with treatment planning from this study paves the way for more targeted approaches to safety improvement.

QA errors are prominent in the field of radiotherapy and their association with decision‐type errors is valuable to meaningful improvement. Without a specific consideration of human contributing factors, suggested mitigation strategies from other studies include enhanced communication about known errors, data‐driven protocol development, and funding for safety tools that balance advancing technological complexity.^20^ The finding from this study, that decision errors are largely involved with QA‐type errors, supports a focus on education and training. The recognized knowledge deficit behind QA errors again allows for a more targeted and effective path toward improved safety.

While aviation and other industries differ significantly in many ways from radiation therapy, human nature, and human contributing factors to safety events were expected to be fairly similar. Our finding that supervisory failures lead to unsafe acts was, therefore, not surprising. Associations between human factors and radiotherapy‐specific error types were not as well anticipated. Findings do align with a common‐sense understanding of the treatment planning and QA processes, but the results of this study point to safety improvement pathways that were not necessarily expected; the nature of radiotherapy safety, risk, and human interactions is so complex that very specific safety improvement strategies are not typically obvious. Study findings did, therefore, satisfy the goal of this work which was to find meaningful predictive patterns of human factors as they relate to radiotherapy safety events.

While the goals of this research were met, our study had multiple limitations. First, study data was limited by the finite and anonymous nature of the incident learning reports. There was no opportunity to follow up with report submitters and no way to determine whether certain countries or even departments made up a disproportionate share of the data sample. This study was also restricted to events of higher severity yet lower severity events are typically more common. The inclusion of such data in future work would likely add to the statistical significance of the results. Last, while well‐defined protocols were used to minimize investigator bias, this study was unavoidably subject to some amount of influence. Given the inherent nature of a large international incident learning database, these limitations are reasonable and do not obfuscate the value of our findings. Additional work with an added qualitative component (follow up with event submitters), with lower severity events, and with a larger sample size would be advantageous and could further substantiate the results from this work.

## 5 CONCLUSIONS

An analysis of incident learning data through a human factors perspective revealed significant associations that can be used to inform future safety improvements. Safety culture and risk mitigation can be enhanced through efforts at the supervisory level. Improved supervision with respect to training, education, and a commitment to best practice standards will reduce the number of unsafe acts that take place. Treatment planning errors are associated with skill‐based errors (mistakes made without a great deal of thought while performing familiar tasks). This highlights a need to increase mindfulness as part of treatment planning. While increased automation has contributed to safety improvements in general, a balance needs to be struck between automation and the human thought‐based component of planning. Errors made performing QA work are associated with decision‐type errors where there is a lack of knowledge or skill. Effective training and education are therefore important for mitigating this risk. These suggested strategies for safety improvement are solidly based on reported experiences in the field and are therefore likely to be effective.

## AUTHOR CONTRIBUTIONS

Sheri M. Weintraub is the corresponding author who led the study design, data acquisition, manuscript drafting, and revisions. B.J. Salter contributed to the study design, as well as the manuscript content, analysis, and revisions. C.L. Chevalier contributed to the study design as well as the manuscript content, analysis, and revisions. S. Ransdell contributed to the study design, statistical analysis, and manuscript content, analysis, and revisions.

## CONFLICT OF INTEREST

Dr. Sheri M. Weintraub reports personal fees from ASTRO Radiation Oncology Healthcare Advisory Council, outside the submitted work; and Member of Work Group RO‐ILS. Dr. B. J. Salter reports personal fees from ASTRO RO‐HAC Committee, during the conduct of the study. The rest of the authors declare to have no conflict of interest.
